# Altered engagement of autobiographical memory networks in adult offspring of postnatally depressed mothers

**DOI:** 10.1016/j.biopsycho.2016.05.006

**Published:** 2016-07

**Authors:** Birthe Macdonald, Lynne Murray, Christina Moutsiana, Pasco Fearon, Peter J Cooper, Sarah L. Halligan, Tom Johnstone

**Affiliations:** a Department of Psychology, University of Reading, Whiteknights, Reading RG6 6AL, United Kingdom; b Department of Psychology, Stellenbosch University, Private Bag X1, Matieland, Stellenbosch 7602, South Africa; c Research Department of Clinical, Educational and Health Psychology, University College London, 1-19 Torrington Place, London WC1E 7HB, United Kingdom; d Department of Psychology, University of Bath, Claverton Down, Bath BA2 7AY, United Kingdom; e Department of Psychiatry and Mental Health, University of Cape Town, J-Block, Groote Schuur Hospital, Observatory, Cape Town, South Africa; f Department of Psychology, School of Social & Behavioural Sciences, University of Kingston, Penrhyn Road, Kingston upon Thames, KT1 2EE, UK

**Keywords:** Autobiographical memory, Postnatal depression, Emotion, Regulation, Prefrontal cortex, Development, Risk, Executive function

## Abstract

Maternal depression is associated with increased risk for offspring mood and anxiety disorders. One possible impact of maternal depression during offspring development is on the emotional autobiographical memory system.

We investigated the neural mechanisms of emotional autobiographical memory in adult offspring of mothers with postnatal depression (N = 16) compared to controls (N = 21). During fMRI, recordings of participants describing one pleasant and one unpleasant situation with their mother and with a companion, were used as prompts to re-live the situations. Compared to controls we predicted the PND offspring would show: greater activation in medial and posterior brain regions implicated in autobiographical memory and rumination; and decreased activation in lateral prefrontal cortex and decreased connectivity between lateral prefrontal and posterior regions, reflecting reduced control of autobiographical recall.

For negative situations, we found no group differences. For positive situations with their mothers, PND offspring showed higher activation than controls in left lateral prefrontal cortex, right frontal pole, cingulate cortex and precuneus, and lower connectivity of right middle frontal gyrus, left middle temporal gyrus, thalamus and lingual gyrus with the posterior cingulate.

Our results are consistent with adult offspring of PND mothers having less efficient prefrontal regulation of personally relevant pleasant autobiographical memories.

## Highlights

• We studied young adult offspring of mothers with postnatal depression (PND).
• We examined prefrontal regulation of autobiographical memories involving the mother.
• PND offspring differences were only found for recall of pleasant memories.
• There was reduced prefrontal connectivity with posterior cingulate and precuneus.
• This mechanism might increase risk for psychopathology in offspring of PND mothers.

## 1 Introduction

Parental depression is widely recognized as a major risk factor for affective disorders in offspring (Beardslee, Keller, Lavori, Staley, & Sacks, 1993; Nomura, Wickramaratne, Warner, Mufson, & Weissman, 2002; Weissman et al., 1986). If the offspring of depressed parents have a higher risk of developing depression, they might be expected to show some of the features and behaviours observed in patients diagnosed with clinical levels of depression. In this study, we examined one such function that is affected by depression, the processing of emotional autobiographical memories. Autobiographical memory is crucial in the development of an individual's personality, and provides a reservoir of experience which prospectively influences interpretation of, and responding to, events. It is inextricably related to social functioning, providing a record of previous social encounters and the content for much social conversation, serving to guide social interactions (Bluck, Alea, Habermas, & Rubin, 2005; Holland & Kensinger, 2010). Considerable research has demonstrated that emotional, personally-relevant life events can be recalled more vividly than less emotional or relevant memories (Holland & Kensinger, 2010), which might reflect the impact of such events at the encoding and consolidation stages. Further research has highlighted a role for emotional autobiographical memories in regulating emotions (Holland & Kensinger, 2010), with the ability to remember the specifics of positive emotional episodes serving to attenuate negative emotional responses to aversive situations (Cooney, Joormann, Atlas, Eugène, & Gotlib, 2007).

Consistent with this, there is increasing evidence that autobiographical memory is impoverished in major depression (Dalgleish & Werner-Seidler, 2014). Paradigms that elicit autobiographical recall have established that individuals with major depression are less able than healthy individuals to detach themselves from memories of negative events (Kuyken, Watkins, Holden, & Cook, 2006; Nolen-Hoeksema, 2000) or retrieve specific autobiographical memories (Williams et al., 2007). Both of these phenomena are exacerbated by the tendency to engage in ruminative processing which is characteristic of the disorder (Watkins & Teasdale, 2001), and have been linked to impairments in executive control (Dalgleish et al., 2007). Hence, the preferential and perseverative processing of negative information appears to be one aspect of autobiographical memory that characterises depressed individuals and those at risk for depression (Gibbs and Rude, 2004, Kuyken and Dalgleish, 2011, Rawal and Rice, 2012).

Studies have also highlighted impairments in positive or pleasant autobiographical memories in depression or those at elevated risk for depression. One prominent feature of depression is anhedonia, a loss of the ability to gain positive experience and pleasure from experiences previously enjoyed (Cryan and Holmes, 2005, Treadway and Zald, 2011). Consistent with this, there is evidence that positive memories are less vivid and intense in recovered depressed than in never-depressed individuals (Werner-Seidler and Moulds, 2011, Werner-Seidler and Moulds, 2012), and that this deficit may impinge on individual ability to repair dysphoric mood (Joormann & Siemer, 2004; Joormann, Siemer, & Gotlib, 2007). Thus, while the tendency to retrieve overgeneral autobiographical memories has been explained as an attempt to avoid extreme levels of emotion when the event in question is negative (Holland & Kensinger, 2010), it may also diminish the emotion regulatory potential of specific positive memories (Cooney et al., 2007).

A small number of studies have examined the neural basis of disturbances in the autobiographical recall of emotional memories in depression and those at risk for depression, and have shown altered activation in brain regions central to autobiographical memory. The retrieval of emotional autobiographical memories involves the engagement of anteromedial temporal lobe memory systems, as well as lateral prefrontal cognitive executive systems involved in the strategic activation (or inactivation) of long-term representations of information (LaBar & Cabeza, 2006), and in elaboration and appraisal of their significance (Joormann and Siemer, 2004, Rawal and Rice, 2012). In addition, a network of cortical midline structures including medial frontal gyrus, anterior cingulate cortex (ACC), posterior cingulate cortex (PCC) and precuneus, is thought to be involved in imagery and self-referential processing, which reflects the “re-living” component of personal memory retrieval (Summerfield, Hassabis, & Maguire, 2009). In an investigation of specific *vs.* general memories in depressed compared to non-depressed participants, activation differed in hippocampus and parahippocampal gyrus as well as anterior insula, which the authors suggested might reflect over-general memory in depression (Young et al., 2012). Reduced activation in ventrolateral prefrontal cortex and cuneus has been observed in remitted depressed participants while attempting to repair sad mood using positive autobiographical memories, with lower activation in those regions predicting the subsequent return of symptoms (Foland-Ross, Cooney, Joormann, Henry, & Gotlib, 2014). Compared to healthy controls, depressed patients showed reduced activation in prefrontal regions while they attempted to distinguish between stimuli related or unrelated to their own experience, a pattern of neural activation consistent with their inability to inhibit irrelevant autobiographical information (Whalley, Rugg, & Brewin, 2012). Based on these few studies, there is initial evidence for disrupted functioning of the neural regions that regulate the quality of autobiographical memory in depressed patients, as well as patients in remission. Further systematic investigation of the relationship between risk for depression and the neural correlates of autobiographical memory for both positive and negative events is called for.

The current study investigated the neural mechanisms involved in emotional autobiographical memory in offspring of mothers who suffered from postnatal depression (PND). The sample was derived from the Cambridge Longitudinal Study which has followed up infants whose mothers suffered from PND and healthy controls through to young adulthood (e.g. (Halligan, Murray, Martins, & Cooper, 2007; Murray, 1992; Murray et al., 1999, Murray et al., 2011). Data from this sample have provided evidence that autobiographical memory for emotional events might be altered in individuals at risk for depression (Murray, Halligan, Adams, Patterson, & Goodyer, 2006). The current assessments were completed when offspring were 22 years of age (i.e. 22 years after their mother’s postnatal depressive episode). In the current study we used a script-driven imagery based approach to examine the neural circuits associated with autobiographical memory recall. Considering participants’ qualitatively different experiences of their mothers due to presence/absence of PND, as well as previously reported differences in peer relationships (Murray et al., 1999, Murray et al., 2006), one positive and one negative situation each with their mother and another person were chosen for this investigation. These descriptions were recorded and played back to participants during an fMRI session and participants were subsequently asked to mentally re-live that situation. We compared brain activation during this re-living phase between offspring of mothers with PND *vs.* the offspring of mothers without PND (controls). We expected to find differential activation in areas implicated in autobiographical memory, including reduced activation in lateral prefrontal cortex, and increased activation in medial prefrontal cortex, ACC, PCC and precuneus. We further hypothesized that connectivity between lateral frontal control regions and more medial and posterior memory regions would be reduced in PND offspring compared to controls, reflecting a lack of prefrontal control of autobiographical recall (Dalgleish et al., 2007). We investigated whether any such differences were evident when re-living negative experiences, positive experiences, or both.

## 2 Method and materials

This study was reviewed and approved by the Oxfordshire NHS and University of Reading Research Ethics committees. Participants provided written, informed consent prior to taking part.

### 2.1 Participants

The sample was originally recruited at 2 months postpartum, through screening a community sample of primiparous mothers of healthy, full-term infants for PND (31). The Edinburgh Postnatal Depression Scale (EPDS) (Radloff, 1977) was administered at 6 weeks postpartum, and women scoring over 12 were interviewed; 61 women who met research diagnostic criteria for depressive disorder were identified, 58 of whom were recruited for the study. Forty-two non depressed mothers were also recruited via random selection from the same postnatal population. The young adults in this study were the infants in the original post-partum sample. At 22-years of age, 45 (76%) offspring of mothers with PND (PNDO group) and 39 (93%) offspring of mothers without PND (control group) were available for a day-long study visit, during which they completed multiple behavioural and neuroimaging assessments. Of these, 37 (19 male) young adults between 21 and 23 years of age were able to complete the current narrative task, 16 from the PNDO group (exclusions were due to diabetes, epilepsy, metal implants, and lack of available scanning time for completion of all scanning tasks).

### 2.2 Narrative task

We used a script-driven imagery based task, which was derived from studies of posttraumatic stress disorder. In its original form, script driven imagery requires participants to note down full details of a specific autobiographical event, including emotional and sensory components (Shin et al., 1999). Descriptions are then edited by an experimenter to a length of approximately 100 words, and used as a cue for recall during neuroimaging. In the current study, participants were asked to recollect and describe four different situations prior to scanning: One negative and one positive situation involving each of their mother and another companion (i.e. mother/positive, mother/negative, other/positive, other/negative). Previous research has shown effects of PND on the mother-child relationship (Murray et al., 2006). Thus, to assess the degree to which observed differences were specific to recollections of interactions with mothers, we used the recollection of a past experience with a companion as a within-subjects comparison condition. Participants were asked to talk about each situation in the first person and to include as much detail as possible, including emotions and bodily sensations. These narratives were recorded and a 30-s segment was generated for each, with standardised mean sound intensity, for use in the subsequent fMRI scanning task.

The experimental task was a block design with two 30-s blocks for each condition (mother/positive, mother/negative, other/positive, other/negative): listening and imagining, followed by 30 s of rest before the next listening block. Before each block, a 1-s instruction was given, consisting only of the words “Listen”, “Imagine” or “Relax”. The four conditions were counterbalanced across subjects to control for order effects. Instructions (see supplementary information) for the task were presented using EPrime 2.0 software (Psychology Software Tools, Pittsburgh, PA) on a PC connected to an MRI compatible goggle system (NordicNeuroLab AS, Bergen, Norway) mounted on the head coil. Recorded stimuli were presented through the same PC, connected to a set of MRI-compatible electrostatic headphones (NordicNeuroLab AS, Bergen, Norway).

### 2.3 Narrative ratings

Subsequent to recording the narratives, participants were asked to rate how positive or negative, and how arousing the situation they had just talked about had been. They scored this on two scales from −50 (*extremely negative* or *extremely relaxed*) to 50 (*extremely positive* or *extremely activated*). The same questionnaire was given to them following scanning to score how activated/relaxed and positive/negative they rated themselves whilst imagining these situations.

### 2.4 Other possible explanatory measures

To assess the possible impact of past depressive episodes in offspring (assessed at 8, 13 16 and 22 years by the Structured Clinical Interview for DSM-IV, SCID and coded present/absent) (First, 2005) and current depressive symptoms (Centre for Epidemiological Studies Depression Scale; CES-D) (Radloff, 1977) as well as gender, these variables were included in a follow-up analysis. Because attachment has been found to affect the processing of emotional information in this sample (Moutsiana et al., 2014), participants’ infant attachment at 18 months (assessed using the Strange Situation (Ainsworth, Blehar, Waters, & Wall, 2014; Blehar, Waters, & Wall, 1978), coded secure/insecure) was used as an additional regressor. Maternal depression was also assessed via clinical interview at multiple time points through the course of the study, generating an index of total months of maternal depression during their child’s lifetime. This was included in analyses as a regressor to assess whether any findings might be explained by chronicity of maternal depression.

### 2.5 FMRI data acquisition

Structural and functional data were collected on a 3T Siemens Trio MRI scanner with 12 channel head matrix coil (Siemens, Malvern, PA) at The University of Reading Centre for Integrative Neuroscience and Neurodynamics (CINN). Functional scans consisted of a t2*-weighted gradient echo, EPI sequence (30 interleaved transverse slices, phase encoding P to A, 4 mm thickness, 1 mm interslice gap; 64 × 64 matrix; 192 mm field of view; TR: 2000 ms, TE: 30 ms, Flip Angle: 90°; 212 whole-brain volumes). A high resolution whole-brain three dimensional structural image was also acquired using an MPRAGE sequence with 176 × 1 mm slices. (1 × 1 × 1 voxels size, TE: 2.52 ms, TR: 2020 ms, TI:1100 ms, FOV: 250 mm, Flip Angle: 90°).

### 2.6 FMRI data analysis

The data were processed using FSL (Jenkinson, Beckmann, Behrens, Woolrich, & Smith, 2012). MCFLIRT (Jenkinson, Bannister, Brady, & Smith, 2002) was applied to correct for motion. The functional time series were high-pass filtered using a 100 s cut off. Images were spatially smoothed using a 5 mm full width at half maximum Gaussian kernel filter.

#### 2.6.1 Single subject analysis

Individual participant data were analysed with a general linear model with separate regressors for each of the eight one-second instructions (“listen” and “imagine” for four different conditions) as well as for the eight 30-s blocks of listening to and the eight 30-s blocks of imagining the different scenarios. These regressors were created by convolving a stimulus boxcar function with the standard FSL double gamma function. Motion estimates were added as regressors to control for uncorrected head displacement.

Contrast analysis was focused on the imagination phases of the experiment. The primary contrasts of interest compared responses to the narratives concerning their mother to those evoked in the peer companion control condition separately within positive and negative conditions (i.e. mother/positive vs other/positive, and mother/negative vs. other/negative).

Registration to a standard space was performed using a two stage procedure with FLIRT (Jenkinson & Smith, 2001). The mean functional volume for each participant was registered to the individual’s high resolution structural image using a 6 ° of freedom (DOF) rigid body transformation. In a second step the individual’s high resolution structural image was normalised to the Montreal Neurological Institute (MNI) template brain using a 12 DOF affine transformation. These two transformations were combined and used for subsequent registration of that participant's contrast images to MNI space before higher-level group analysis.

#### 2.6.2 Psychophysiological interaction (PPI) connectivity analysis

In order to determine connectivity across the whole brain, we selected the clusters which showed the most significant group activation differences, extracted each cluster's mean BOLD time series for the task and entered this to the individual-subject level GLM as an additional EV. Four PPI EV’s were created by setting up interactions between the demeaned cluster time series and each condition timing regressor (centred). Contrasts of parameter estimates between these PPI EVs were carried forward into the group level analysis.

#### 2.6.3 Higher level (group) analysis

Comparison of contrasts between groups was carried out using Mixed Effects (FMRIB’s Local Analysis of Mixed Effects, FLAME 1) with automatic outlier de-weighting and Random Field-based cluster thresholding correcting for multiple comparisons to insure a familywise error of p < 0.05. To assess the possible impact of differences in infant attachment (as assessed by the Strange Situation (Ainsworth et al., 2014, Blehar et al., 1978)), and lifetime depressive episodes (SCID; (First, 2005)) and current depressive symptoms (CESD; (Radloff, 1977)), as well as total study months of maternal depression, results for all significant reported clusters were checked by including these variables as regressors (descriptive statistics for these variables are given in Table 1) in a general linear model assessing *unique* variance explained by each explanatory variable (cf. Cohen and Cohen, 1983; Miller & Chapman, 2001), separately for the activation and PPI clusters.

## 3 Results

### 3.1 Behavioural results

Subjective ratings of valence and arousal of how subjects felt both at the time of each scenario, and while imagining in the scanner are shown in Table 2. We tested for group differences in ratings using mixed-effects ANOVA with scenario valence (positive, negative) and scenario person (mother, other) as within-subjects factors and group as a between-subjects factor. There were no main effects of group, nor any interaction effects including group, for ratings of how the situations felt at the time. There was a marginally significant interaction of group by valence, *F*(1,32) = 4.1, *p* = 0.051) for how subjects felt while imagining the scenarios; the difference in rated valence between positive and negative scenarios was significantly greater for control group than for PND offspring. There were no other effects involving group.

### 3.2 Group differences in brain activation and connectivity

**Fig. 1:**
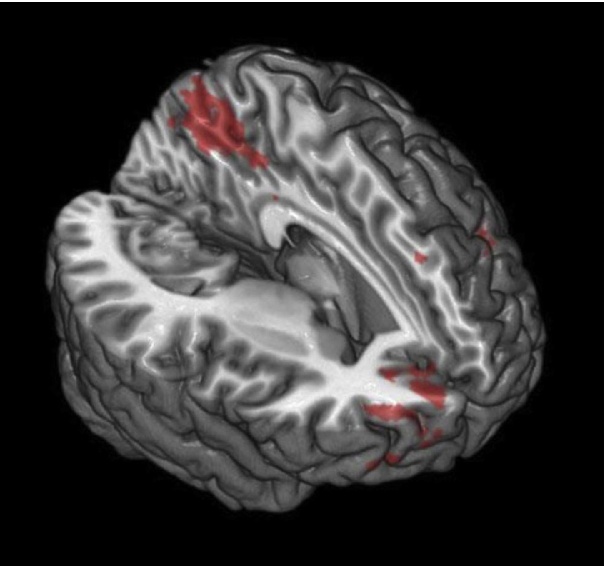
Group x condition interaction resulting from the mother/positive – other/positive comparison. The graph depicts this difference in precuneus, illustrating significantly lower activation in other/positive than mother/positive scenarios in the PND offspring group. FWE < 0.05.

Contrary to hypotheses, for negative scenarios involving the mother (compared to other), we found no significant group differences in brain activation. For positive scenarios, PND offspring showed significantly higher activation for this sample contrast than the control group in a number of regions including left lateral prefrontal cortex, right frontal pole, cingulate cortex and precuneus (see Fig. 1, Table 3a). This was due to less activation during mother positive scenarios (compared to companion) in the control group (*F*(1,20) = 7.14, *p* = 0.015), but marginally greater activation to positive mother than positive companion scenarios in the PND group (*F*(1,15) = 3.53, *p* = 0.08).

**Fig. 2:**
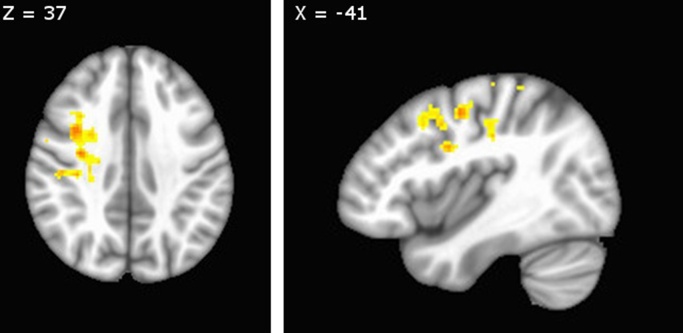
Differences in connectivity in the mother/positive – other/positive contrast, using the precuneus as a seed region. The graph (depicting this effect in MFG) shows the direction of the group difference – for PND offspring connectivity is significantly lower in mother/positive scenarios than other/positive. FWE < 0.05.

A PPI analysis on the mother/positive – other/positive contrast using the most activated cluster within the precuneus/posterior cingulate as a seed region revealed group differences in connectivity with the right middle frontal gyrus (MFG), left middle temporal gyrus (MTG), thalamus and lingual gyrus (see Fig. 2, Table 3b). Post-hoc *t*-tests revealed that PND offspring showed reduced connectivity between these regions and the seed region during the positive situations involving their mothers compared to those involving their companion. In contrast, controls showed no significant difference in connectivity between these conditions.

These observed group effects in activation (*F*(1,28) = 7.4, *p* = 0.011) and PPI connectivity (*F*(1,28) = 14.4, *p* = 0.001) persisted when gender, and differences in infant attachment, lifetime depressive episodes, current depressive symptoms and total study months of maternal depression, were included as regressors in followup general linear models assessing unique variance explained. None of the additional regressors showed a significant association with activation (all p > 0.15) or PPI connectivity (all p > 0.33), indicating that the PND group effects in this sample could not be statistically accounted for by attachment status nor current or past depression in the offspring or total months depression in the mother.

## 4 Discussion

We examined neural responding in the offspring of postnatally depressed mothers compared to control group offspring during re-living of past emotional episodes involving their mothers, relative to episodes involving a companion. We hypothesised differential activation in a network consisting of midline and inferior temporal brain regions involved in self-referential processing and episodic autobiographical memory, and lateral prefrontal regions involved in selective memory retrieval, elaboration and reappraisal.

For positive memories, but not for negative memories, comparison of mother-related vs. other-related scenarios revealed a number of cortical midline structures which were more strongly activated by PND offspring during the mother/positive than during the other/positive condition. Controls showed no significant activation difference between the two scenarios. In particular, a large midline cluster spanning the precuneus and posterior cingulate showed this group activation difference. These brain regions have been linked to the re-living of autobiographical memory, specifically, its vividness and the amount of detail that is remembered (LaBar & Cabeza, 2006), which may reflect the qualitative difference in the two groups’ memories of their childhood experience.

Furthermore, a PPI connectivity analysis of the mother/positive versus other/positive contrast using this posterior midline cluster as a seed region revealed a network of brain regions that showed differences between groups, including thalamus, middle temporal gyrus and middle frontal gyrus. Again, whereas control participants tended to show similar levels of connectivity between posterior cingulate/precuneus and these regions during both scenarios, PND offspring showed decreased functional connectivity during mother/positive compared to other/positive scenarios.

This pattern of increased activation but decreased connectivity between frontal and posterior midline regions during attempts to relive past positive experiences with their mothers is consistent with a model in which adult offspring of PND mothers recall personally relevant autobiographical memories in a less efficient manner. Although there is active debate about exactly how the autobiographical memory system functions, a predominant current view is that autobiographical memories are reconstructed, that is, pieced together, from a selection of stored information, rather than being stored as intact cohesive memories of events. This process is regulated by the PFC, which serves to activate relevant and deactivate irrelevant memories (Conway & Pleydell-Pearce, 2000). Thus although the original encoding of events in autobiographical memory might largely be an automatic process, the retrieval and elaboration of memories often requires executive processing in order to activate the constituent information and assemble a coherent memory. This process is subject to biases (in what is activated, the weighting given to the various components of the memory, and the manner in which the components are assembled) and is susceptible to interference from competing memories or cognitive demands. There is some evidence that those with current or previous depression show biases in autobiographical memory recall, and are less able to retrieve autobiographical memories, particularly positive memories, with the same vividness and intensity as never-depressed individuals. Such deficits could occur at the encoding or recall stage, although the little available evidence suggests that it is efficacy in recall that is diminished, because the intensity and vividness of recalled positive autobiographical memories is only diminished during sad mood, not in the absence of sadness (Werner-Seidler & Moulds, 2011).

Prefrontal executive control brain regions are thought to be instrumental in appropriately activating previously stored information during recall, a process that includes the prevention of unwanted memory intrusions and self-reflective rumination. This type of effective regulation of personally-relevant memories is vital for sustained emotional wellbeing (Gross and Muñoz, 1995, Joormann, 2010); a compromised ability to regulate the content and time course of episodic memories is a hallmark of rumination in major depression (Joormann & Gotlib, 2010). In this study, there was a relative de-coupling of prefrontal brain regions with the precuneus and posterior cingulate observed in PND offspring during recollection of past positive experiences with their mothers, though there was no corresponding decrease in PFC activation. This is more consistent with less efficient prefrontal regulation of autobiographical memory recall, rather than a lack of prefrontal activity overall. Thus, PND offspring may have found it more cognitively effortful to select the positive memories relating to their mothers from a set of competing memories.

The specificity of the observed group differences to positive memories involving the mother but not another companion argues against a purely biological explanation for these findings. Instead, the development of neurobiological systems for encoding and recall of emotional memories in offspring of mothers with PND may be particularly vulnerable to social and environmental influences, such as impaired social interaction between mother and child and associated acute early-life stress. Those brain regions with a high density of glucocorticoid receptors and that have a slower postnatal developmental profile, such as the lateral PFC, are thought to be more susceptible to such effects (Pechtel & Pizzagalli, 2011). Nonetheless, our findings might also reflect underlying genetic vulnerabilities shared between the mother and child that only become manifest over the course of development and in specific contexts (van Oostrom et al., 2012). Twin studies of the structural properties of different brain regions have shown that heritability increases with age, with the implication that brain regions that develop later in life (such as the lateral PFC) also show a greater influence of genetics later in life (Lenroot et al., 2009). Such findings are consistent with an increasing awareness of the role of genetics in children’s socioemotional development over time (Fearon, Shmueli-Goetz, Viding, Fonagy, & Plomin, 2014).

Some limitations must be taken into account when considering the results from this study. Inevitably, due to the study’s longitudinal nature, a number of participants from the original sample did not provide data. In addition, narrative tasks such as that used in this study, while having ecological validity, allow limited control over the content of the narratives. It is plausible that reduced availability or vividness of positive memories with their mothers might underlie the neural effects observed in this study, but we have no evidence relating to this. Mothers in the PND group were more likely than control group mothers to have further depressive episodes subsequent to the postnatal period (Murray et al., 2006), which might have lead to a relative impoverishment of offspring’s positive memories with their mother. Nonetheless, including duration of mother depression during the child’s lifetime as a regressor in the analysis had no impact on the observed patterns of neural activation and connectivity.

Despite such limitations, the fact that altered neural functioning at age 22 was associated with maternal PND suggests long-lasting alterations in systems that allow for effective recall of emotional autobiographical memories. In general, lower prefrontal efficiency in regulating positive autobiographical memories might be one of the factors that render offspring of PND mothers at elevated risk for developing depression. It is an intriguing question whether such risk might be mitigated by a psychological intervention that targeted executive control of autobiographical memories. Nonetheless, conclusions on this point would be premature in the absence of more definitive evidence relating to the functional significance of the pattern of neural alterations that we observed, which need not necessarily be maladaptive. Given the complexity of possible interactions between genetic, neurobiological, social and behavioural factors during development, larger scale longitudinal neuroscience studies including measurement of the parent-child relationship throughout development will be necessary to identify the processes that give rise to such effects observed in young adulthood.

## Disclosure

Pasco Fearon received an honorarium for giving a workshop on attachment at the Meeting of Minds Conference, 2014, London, organised by Shire Pharmaceuticals. There are no other biomedical financial interests or potential conflicts of interest.

## Figures and Tables

**Table 1:** Descriptive statistics of covariates for offspring depression and attachment, and total maternal depression.

Variable		PND	Controls	Statistic
Offspring Depression				
Lifetime Depression	Present	7	4	χ^2^ = 2.65, df = 1, p = 0.10
Absent	9	17
Current depressive symptoms (CESD)	Mean	7.8	10.6	*t* = 0.86, df = 35, *p* = 0.39
SD	9.4	10.5
Offspring Attachment				
18 month attachment (Strange Situation)	Secure	7	15	χ^2^ = 2.89, df = 1, p = 0.089
Insecure^a^	9	6
Maternal depression				
Total study months of maternal depression	Mean	30.4	8.6	*t* = 3.19, df = 35, *p* = 0.003
SD	26.0	15.3

CESD: Centre for Epidemiological Studies Depression Scale.

a Insecure group comprises: avoidant n = 12, ambivalent n = 1, disorganised n = 2.

**Table 2:** Subjective ratings of the experience of positive and negative scenarios at the time, and the experience during imagining the scenarios, reported for PND and control group offspring.

		positive situation				negative situation			
		with mother		with other		with mother		with other	
		valence	arousal	valence	arousal	valence	arousal	valence	arousal
(a) at the time									
Control	Mean	30	−8	36	1	−21	25	−23	29
	SD	10	31	14	35	22	17	29	19
PND	Mean	19	−6	31	6	−14	21	−15	22
	SD	25	27	14	27	24	17	26	19
(b) while imagining									
Control	Mean	27	−4	29	−2	−14	10	−23	15
	SD	14	27	10	26	13	17	14	24
PND	Mean	22	−18	28	−7	−4	12	−12	11
	SD	17	25	17	27	23	25	23	24

**Table 3:** Results from group analysis of (a) brain activation to mother-positive versus other-positive scenarios, and (b) PPI connectivity with precuneous during mother-positive versus other-positive scenarios. All clusters FWE <0.05.

	% Signal Contrast			Cluster Size		Cluster Center of Gravity		
(a) Mother/positive – Other/positive activation	Control	PNDO	Zmax	voxels	mm3	x	y	z
precuneus and posterior cingulate	−0.07	0.51	3.77	3288	26304	−3	−62	44
left middle frontal gyrus	−0.24	0.23	3.33	241	1928	−42	13	33
left posterior supramarginal gyrus	−0.26	0.18	3	236	1888	−50	−53	26
right frontal pole	−0.36	0.53	3.33	180	1440	25	58	8
right frontal pole	−0.07	0.28	2.76	163	1304	8	51	8
right lateral occipital cortex	−0.28	0.19	2.83	90	720	31	−73	34
left inferior frontal gyrus	−0.29	0.33	2.87	49	392	−44	27	21
left orbitofrontal cortex	−0.38	0.23	3.01	46	368	−22	30	−17
right frontal pole	−1.86	0.33	2.6	36	288	34	61	−6
(b) Mother/positive – Other/positive PPI connectivity								
Right Middle Frontal Gyrus			4.24	948	7584	35	−5	42
Left Middle Temporal Gyrus			4.5	491	3928	−62	−51	−8
Thalamus			3.51	166	1328	−4	−26	4
Lingual Gyrus			2.72	65	520	30	−51	−2
